# Thrombocytosis as an Initial Presentation of Plasma Cell Neoplasm: A Case Report

**DOI:** 10.7759/cureus.4286

**Published:** 2019-03-21

**Authors:** Arslan Naeem, Surabhi Amar, Divyesh Mehta, Mustafa N Malik

**Affiliations:** 1 Internal Medicine, Maricopa Medical Center, Phoenix, USA; 2 Hematology and Oncology, Maricopa Medical Center, Phoenix, USA; 3 Internal Medicine, The University of Arizona, Tucson, USA

**Keywords:** thrombocytosis, plasma cell neoplasm, multiple myeloma, myeloproliferative disorders, essential thrombocythemia

## Abstract

Plasma cell neoplasms are usually associated with normal or decreased platelet count. The association of thrombocytosis and multiple myeloma is exceedingly rare, with only six such cases reported in the literature until now. Differentiating clonal from secondary causes of thrombocytosis can be extremely difficult, yet the distinction has important therapeutic implications. We report the case of a woman presenting with thrombocytosis that led to the diagnosis of multiple myeloma. The possible etiological link between both these entities is also discussed.

## Introduction

Thrombocytosis can be reactive (more common) or a primary myeloproliferative disorder (MPD) which includes essential thrombocythemia. Many inflammatory (celiac disease, inflammatory bowel disease, pancreatitis, iron deficiency), rheumatologic (vasculitis), infectious, or neoplastic conditions cause reactive thrombocytosis. It has been reported with many solid tumors (e.g., lung, stomach, ovarian and renal cancers) as well [1]. Patients with secondary thrombocytosis typically have clinically apparent, coexisting, underlying systemic diseases that account for the elevated platelet count. Unlike patients with secondary thrombocytosis, those with clonal thrombocytosis have thrombotic, vascular, and bleeding complications. Hematological malignancies are usually associated with thrombocytopenia. The association between multiple myeloma and thrombocytosis is infrequent. As far as thrombocytosis is concerned, the appearance of multiple myeloma has been reported in only six instances [2]. We report the case of a woman who had multiple myeloma with associated thrombocytosis. We also reviewed the published data on this association.

## Case presentation

**Figure 1:**
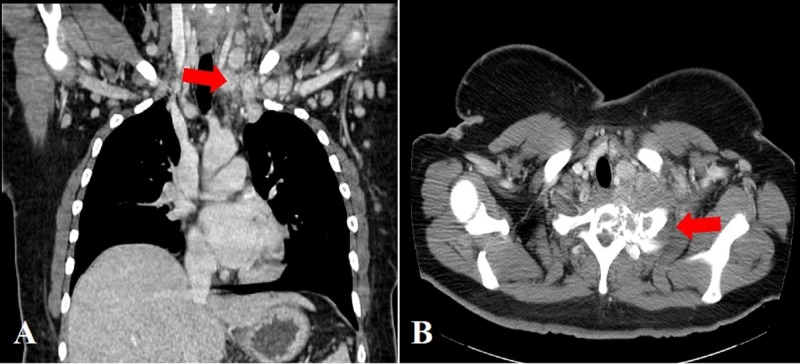
Computed tomography (CT) scan showing A) cervical lymphadenopathy B) 6 x 4.5 cm mass in the para-spinal muscle of the thoracic inlet invading the T1 and posterior part of the first rib with a pathologic compression fracture

A 32-year-old female presented with complaints of fatigue and tingling sensation in extremities. Physical exam was unremarkable without evidence of lymphadenopathy or hepatosplenomegaly. Laboratory findings were significant for hemoglobin (Hb) at 17.2 g/dL, white blood cell (WBC) count at 9 x 103/µL, and platelets 594x 103/µL. She had no fever, weight loss, joint pains or other systemic symptoms. Work up for thrombocytosis was initiated. Bone marrow biopsy showed mildly hypo-cellular marrow (40%) with normal trilineage hematopoiesis, no evidence of malignancy. Janus kinase 2 (JAK2) exon 12 mutation was negative. One month later, she presented to the emergency department (ER) with left-hand weakness and numbness. Computed tomography (CT) scan showed bilateral cervical chain lymphadenopathy and 6 x 4.5 cm soft tissue mass in the paraspinal muscle of the thoracic inlet invading the T1 and posterior rib with pathologic compression fracture (Figure 1).

**Figure 2:**
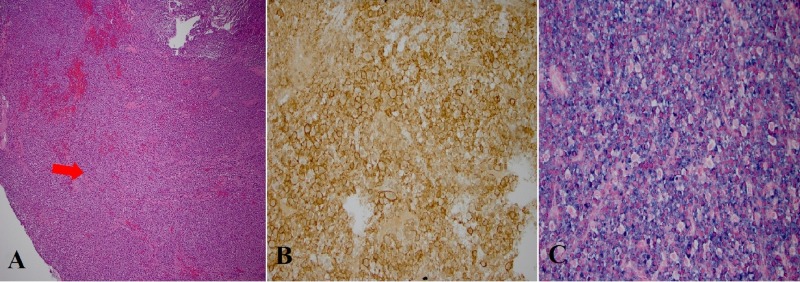
Tissue staining from the open biopsy of the paraspinal mass A) hematoxylin eosinophilin stain showing tumor infiltration B) CD 138 positive immunochemical staining for plasma cells C) staining for lambda restricted plasma cells.

Open biopsy with cervical thoracic fixation from C4-T5 was done. Pathology showed neoplastic infiltration by lambda restricted monoclonal plasma cells. Flow cytometry of the tumor showed 3% lambda restricted plasma cells (Figure 2).

A complete skeletal survey was negative for lytic lesions. Serum protein electrophoresis showed immunoglobulin (Ig) G lambda restricted M spike of 0.2 g/dL. Lactate dehydrogenase (LDH) was normal. Beta-microglobulin level was 2.7 mg/L. Positron emission tomography (PET) scan showed lytic lesions in her iliac bones and sacrum. A diagnosis of multiple myeloma was made and Revlimid/Velcade/Dexamethasone (RVD) regimen was given. Following treatment, her platelet count became normal at 275 x 103/µL. She had a repeat bone marrow biopsy and it was again normal with negative calreticulin (CALR) gene mutation, negative fluorescence in situ hybridization (FISH) for myeloma and MPDs and normal cytogenetics. JAK 2 mutation analysis was positive. The patient does not have any primary bone marrow fibrosis. She went on to have an autologous stem cell transplant and is currently on maintenance Revlimid therapy.

## Discussion

Thrombocytosis is typically discovered as an incidental laboratory finding during routine workup. However, when found, it creates an important diagnostic challenge. In a study involving 280 hospitalized patients with platelet counts of one million per cubic millimeter or higher, 82% (231 patients) had secondary thrombocytosis, 14% (38 patients) had an MPD while only 4% (11 patients) had thrombocytosis of uncertain cause [3]. In another study including 732 patients with platelet counts of 500,000 per cubic millimeter or higher, 88% (643 patients) had secondary thrombocytosis; the most frequent underlying causes in these patients were tissue damage during major surgery, chronic inflammation, infection and carcinoma [4].

Thrombocytosis can be a paraneoplastic manifestation of malignancy. Myeloma has been reported in cases of MPDs causing thrombocytosis. POEMS syndrome (polyneuropathy, organomegaly, endocrinopathy, monoclonal protein, and skin changes) can be associated with thrombocytosis. In the absence of underlying MPD or POEMS syndrome, myeloma is usually associated with normal or low platelet counts [5-6]. It has been suggested that the cytokine interleukin-6 (IL-6), an important component in the pathogenesis and progression of multiple myeloma may provide a common link between multiple myeloma and thrombocytosis. IL-6 is a potent human myeloma-cell growth factor as well as a promoter of megakaryocytopoiesis [7-8]. However, the exact mechanism still remains unclear; other pathogenetic mechanisms need to be explored. The association of multiple myeloma, a lymphoproliferative neoplasm, with thrombocytosis, though documented, is extremely rare [9]. To date, only six cases of myeloma associated with thrombocytosis have been reported [5-6,10] (Table 1).

**Table 1:** Report of cases of multiple myeloma associated with thrombocytosis g: gram; Ig: immunoglobulin; ml: milliliter; µL: microliter; N.R: not reported; Ref: references.

Patient	Sex	Bone Marrow Biopsy	Protein (g/100ml)	Type of M component in serum	Platelet Count (/µL)	Ref.
1	Male	60% plasma cells	Elevated	N.R	800,000	[6]
6	Male	6% plasma cells with otherwise normal marrow	4.99	IgG-Gamma	400-780,000	[6]
3	Male	90% plasma cells	6.2	N.R	400-871,000	[6]
4	Male	65%-70% plasma cells	Elevated	IgD	931-984,000	[5]
5	Male	10% plasma cells	2.25	IgA-Kappa	980-1420,000	[10]
6	Female	30%-40% plasma cells	N.R	IgG-Kappa	1220,000	[10]

The challenge of correctly identifying the etiology of thrombocytosis in a patient becomes crucial when the clinician has to decide treatment options. It is critical to identify the etiology of thrombocytosis even when it is clinically inapparent so that treatment can be directed to the underlying disorder [1]. Work up for thrombocytosis should exclude autoimmune disorders, chronic inflammatory disorders, infections, and iron deficiency. In the absence of MPD, underlying malignancy should always be ruled out in patients found to have unexplained thrombocytosis. Although not yet clinically applicable, development of clonality assays and tests for c-Mpl expression in megakaryocytes and platelets promises to provide diagnostic tools to differentiate clonal from secondary causes of thrombocytosis [1,11].

## Conclusions

Patients with multiple myeloma usually present with thrombocytopenia. However, thrombocytosis can be an initial presentation. Before ascribing thrombocytosis to a clonal cause (myeloproliferative disorder), which is a diagnosis of exclusion and has different therapeutic implications, secondary causes of thrombocytosis must be ruled out.
